# Impact of Power Transformer Oil-Temperature on the Measurement Uncertainty of All-Acoustic Non-Iterative Partial Discharge Location

**DOI:** 10.3390/ma14061385

**Published:** 2021-03-12

**Authors:** Vladimir Polužanski, Nenad Kartalović, Boško Nikolić

**Affiliations:** 1 School of Electrical Engineering, University of Belgrade, 73 Kralja Aleksandra Boulevard, 11120 Belgrade, Serbia; nbosko@etf.bg.ac.rs; 2 Electrical Engineering Institute of Nikola Tesla, University of Belgrade, 8a Koste Glavinica Street, 11040 Belgrade, Serbia; nenad.kartalovic@ieent.org

**Keywords:** partial discharge, acoustic emission, power transformer, transformer oil temperature, measurement uncertainty, nonlinearity, monte carlo filtering, metrology, software

## Abstract

In this paper, the influence of the variation in transformer oil temperature on the accuracy of the all-acoustic non-iterative method for partial discharge location in a power transformer is researched. The research can improve power transformers’ testing and monitoring, particularly given the large transformer oil temperature variations during real-time monitoring. The research is based on quantifying the contribution of oil temperature to the standard combined measurement uncertainty of the non-iterative algorithm by using analytical, statistical, and Monte Carlo methods. The contribution can be quantified and controlled. The contribution varied significantly with different mutual placements of partial discharge and acoustic sensors. The correlation between the contribution and the mean distance between partial discharge and acoustic sensors was observed. Based on these findings, the procedure to quantify and control the contribution in practice was proposed. The procedure considers the specificity of the method’s mathematical model (the assumption that the oil temperature is constant), the non-iterative algorithm’s nonlinearity, and the large variations in transformer oil temperature. Existing studies did not consider the significant effect of the oil temperature on the combined measurement uncertainty of partial discharge location influenced by those phenomena. The research is limited to partial discharge located in the transformer oil.

## 1. Introduction

Power transformers are vital elements of production, transmission, distribution, and industrial utilities. The proper operation and maintenance of power transformers are crucial to avoid unplanned downtime, financial losses, and environmental hazards. Partial discharges are among the most common causes of power transformer failures. The timely and precision detection, monitoring, and diagnostics of partial discharge (PD) improve the reliability of power transformers. In recent years, with significant improvements in sensors, sensor networks, and computing systems (e.g., the industrial internet of things), the implementation of data-driven machine health monitoring models has increased significantly [1].

The all-acoustic method exclusively uses acoustic emission (AE) signals for PD detection in a power transformer. This method has advantages over the other methods for PD detection; it is not affected by electromagnetic interference, can provide information about the PD location, is noninvasive, is simple to install (e.g., to transformers under load), and is suitable for testing and monitoring applications. The disadvantages of this method are its moderate sensitivity (>300 pC), vulnerability to weather conditions (e.g., thunderstorms, rain, and wind), non-PD vibration sources (e.g., loose parts and cooling fans) that interfere with the AE signal, and AE signal attenuation during propagation through different materials (e.g., copper, steel, transformer oil, and water) [2]. Many researchers have demonstrated different approaches for PD localization through the all-acoustic method in a power transformer. The approach based on pattern recognition (lookup time delay vector table) is demonstrated in [3], different approaches employing triangulation based on time-of-flight measurements in [4,5], and the use of a non-iterative algorithm in [6]. More recent approaches are based on intelligent algorithms: genetic algorithms (GAs) [7,8,9], particle swarm optimization (PSO) algorithms [10], artificial neural networks (ANNs) [11], PSO in combination with ANNs [12] or with fuzzy logic [13], and bat algorithms [14]. The most effective results in practice are achieved by the simultaneous use of the acoustic and electrical methods, and the all-acoustic method is only used for the localization of PDs that occur in transformer oil [15].

The non-iterative all-acoustic method was first proposed by Kundu et al. in 2009 [6]. The main advantages of this method are its application possibilities, ease of use, and cost effectiveness [16]. In the field, with appropriate preparation and planning, this method can be applied to transformers in the operating mode using only four acoustic sensors mounted on the transformer wall. The reported experimental results are considered to be acceptable for practical applications; however, newly proposed methods based on intelligent algorithms produce better results in PD detection accuracy. This study aims to further improve the PD localization accuracy of the non-iterative all-acoustic method by addressing another major source of error in PD source location (apart from the time delay calculation) that originates from the method’s mathematical model.

Mathematical model of the all-acoustic non-iterative method is based on the assumption that the velocity of the acoustic signal is constant. The acoustic signal velocity is dependent on the transformer oil temperature [17], i.e., oil temperature is also considered constant. From the prospect of physics, thermodynamic properties of fluids, and transformer construction, this approximation largely deviates from reality; the temperature of the oil is heterogeneous and varies significantly during the transformer operation (depending on the transformer load, environmental temperature, and transformer construction) [18].

In the existing literature, an adopted constant value is used for the value of the AE signal propagation velocity in a non-iterative algorithm. This value may be adopted based on the recommendation for all AE methods to disregard the influence of sound-velocity change and use the constant value of 1413 m/s, which corresponds to the oil temperature of 20 °C [15]. This recommendation originates from the IEEE C57.127-2007 standard, but was removed during the preparation of IEEE C57.127-2018. Some researchers recommend adopting a constant value corresponding to the mean temperature of the transformer oil [19]. Both these recommendations treat the transformer as a homogeneous medium. There is no specific recommendation for the all-acoustic non-iterative method that considers the specificity of the method’s mathematical model, the nonlinearity of the non-iterative algorithm, or the influence of the transformer oil-temperature change (sound-velocity change).

The non-iterative mathematical algorithm yields a solution for a system of nonlinear equations that describe the location of PD. Research on nonlinear dynamics reveals that nonlinear phenomena can yield novel and advantageous effects in many practical engineering problems [20]. The simulation results based on the one-at-a-time method in [21] suggest that the effect of the transformer oil-temperature change on the accuracy of this method is not negligible. In [21], for the cube-shaped transformer with a side length of 0.60 m, the maximum change in the result of PD location (in relation to the nominal value) varies from less than 0.01 to 0.11 m for the same oil-temperature change of 40 °C but different mutual positions of AE sensors and the PD source. A similar result is also reported in [22,23,24,25,26] where different AE sensor, RF, and UHF antenna formations produced different accuracy levels in PD localization.

The research presented in [21] has several shortcomings: the software simulation results were considered only for four selected mutual positions of PD and AE sensors, the contribution of oil temperature to the standard combined measurement uncertainty was not quantified, and the results were not verified experimentally.

In this paper, the procedure to quantify the contribution of oil temperature to the standard combined measurement uncertainty of the non-iterative algorithm is defined. Then, this procedure is implemented as the appropriate assistant software tool, the measurement uncertainty assistant (MUA). In the simulations, for the assumed PD position and the transformer’s dimensions, such positions for AE sensors (and vice-versa) are determined where the influence of the transformer oil-temperature changes on the accuracy of PD location is minimal (optimal). The simulation results are then used to investigate and control the influence of the transformer oil-temperature change on PD localization accuracy (i.e., to observe favorable phenomena, adjust, and plan AE sensors’ placement). Finally, the obtained results are verified experimentally, and an example for using the MUA software in the practice is proposed.

## 2. Materials and Methods

### 2.1. Power Transformer Oil Temperature

The temperature of fluid has a big impact on acoustic signal velocity. In a fluid according to the Newton-Laplace formula, the velocity of sound *c* depends on the adiabatic bulk modulus *K_s_* and density *ρ*.

(1) $$c=\sqrt{\frac{K_{s}}{\rho }}$$

Under ambient pressure, the isothermal bulk modulus *K_T_* (*K_s_* = (*c_p_*/*c_v_*)*K_T_*) can be represented as [27]:

(2) $$K_{0}=K_{00}e^{-\beta_{k}T}$$

where, *K*_00_ is the value of *K*_0_ extrapolated to zero temperature, *β_k_* is the temperature coefficient of the ambient pressure bulk modulus, *c_p_* is the specific heat at constant pressure, and *c_v_* is the specific heat at constant volume. Under ambient pressure for the temperature dependency of the density, a linear approximation can be used:

(3) $$\frac{\rho_{0}}{\rho_{R}}=1-\alpha_{\rho }\;\left(T-T_{R}\right)$$

where, α_ρ_ is the thermal expansivity, subscript *R* denotes a reference state, *p* = 0 (or 0.1 MPa) and *T* = *T_R_* = 273.15 K [27].

Based on Equations (1)–(3), the temperature dependence of the sound velocity can be represented by a linear approximation:

(4) $$c\propto \sqrt{\frac{1+\alpha_{\rho }(T-T_{R})}{1+\beta_{K}(T-T_{R})}}\propto 1-\frac{\beta_{k}-\alpha_{\rho }}{2}(T-T_{R})\;,$$

For mineral oils (β_k_ − α*_ρ_*) > 0 [27], making *c* a decreasing function of the temperature.

The velocity of sound as a physical quantity has complex physical bases theoretically studied and modeled at the molecular level. Mineral oils are of complex composition, and knowledge of *Ks*, *ρ*, and *c* is challenging to determine theoretically, and therefore these quantities are commonly determined by laboratory tests [28,29].

The velocity of acoustic signal propagation in transformer oil depends on the transformer oil temperature. For the typical transformer oil, over the temperature range of −30 °C to 130 °C, the sound velocity rapidly changes from approximately 1600 m/s to 1100 m/s [17]. The velocity also depends on the acoustic signal frequency, and on the gas and water content of the transformer oil. If a high level of precision is to be achieved, it is necessary to consider all the above parameters. However, for many practical purposes, including PD detection and location, an approximation considering only the transformer oil temperature is sufficient [17,30].

### 2.2. Mathemathical Model for Partial Discharge Location

**Figure 1:**
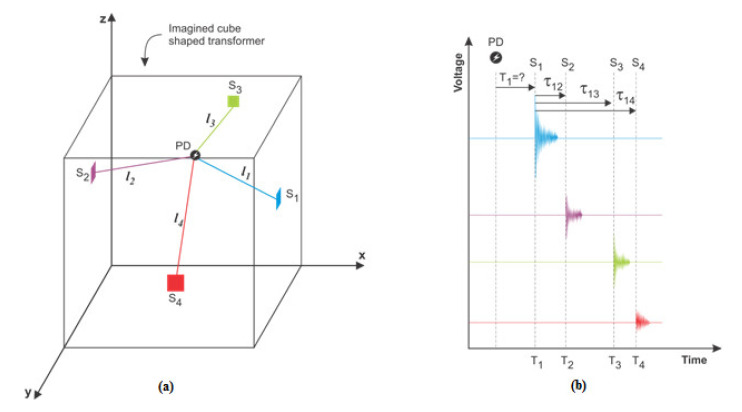
System for detecting the location of partial discharge (PD) by the non-iterative all-acoustic method; (**a**) spatial arrangement of sensors and PD in an imagined cube-shaped transformer; (**b**) appearance of acoustic signals of PD recorded by four acoustic sensors S_1_, S_2_, S_3_, and S_4_.

The mathematical model used for PD localization by the all-acoustic non-iterative method is based on triangulation from GPS localization techniques and the time difference of arrival (TDOA) principle. In the mathematical model, the location of PD is determined indirectly. The input variables affecting the accuracy of PD location are: the velocity of acoustic signal propagation *v*_s_; the PD source coordinates *x*, *y*, and *z*; the sensor coordinates *x*_Si_, *y*_Si_, and *z*_Si_ (*i* = 1,..., 4); and the differences in the time of arrival of AE signals to the sensors, *τ*_12_, *τ*_13_, and *τ*_14_. The time from discharge occurrence to sensor’s S_1_ response, *T*_1_, is unknown. Distances between the PD source and sensors *l*_i_ (*i* = 1, ..., 4) refer to *l*_1_ < *l*_2_ < *l*_3_ < *l*_4_ (Figure 1).

Based on the TDOA principle, solving the system of four non-linear Equations (5)–(8), which is accomplished by the non-iterative mathematical algorithm [6], yields the PD location.

(5) $${\left(x-x_{s_{1}}\right)}^{2}+{\left(y-y_{s_{1}}\right)}^{2}+{\left(z-z_{s_{1}}\right)}^{2}={\left(v_{s}T_{1}\right)}^{2}$$

(6) $${\left(x-x_{s_{2}}\right)}^{2}+{\left(y-y_{s_{2}}\right)}^{2}+{\left(z-z_{s_{2}}\right)}^{2}={\left(v_{s}(T_{1}+\tau_{12})\right)}^{2}$$

(7) $${\left(x-x_{s_{3}}\right)}^{2}+{\left(y-y_{s_{3}}\right)}^{2}+{\left(z-z_{s_{3}}\right)}^{2}={\left(v_{s}(T_{1}+\tau_{13})\right)}^{2}$$

(8) $${\left(x-x_{s_{4}}\right)}^{2}+{\left(y-y_{s_{4}}\right)}^{2}+{\left(z-z_{s_{4}}\right)}^{2}={\left(v_{s}(T_{1}+\tau_{14})\right)}^{2}$$

### 2.3. Procedure for Uncertainty Calculation 

As outlined in Section 2.1, the velocity of acoustic signal propagation in transformer oil depends on the transformer oil temperature.

Because the coordinates of the result of detecting the location of PD (*x*, *y*, *z*) are functions of several variables, and accounting for the correlation between the acoustic signal velocity and the transformer oil temperature, *T*, they are described in a generalized form in Equation (9).

(9) $$g=f_{g}(x,\;y,\;z,\;x_{s_{i}},y_{s_{i}},z_{s_{i}},T,\tau_{12},\tau_{13},\tau_{14}),\;g\equiv x\equiv y\equiv z$$

Standard combined uncertainty for function *f_g_*, *u*_c_(*f_g_*), with no correlation between parameters, where *u*_x_, *u*_s1_, *u*_T_,..., *u*_τ14_ represent the standard uncertainties of respective parameters, is expressed as follows [31]:

(10) $${u^{2}}_{c}(f_{g})={\left(\frac{\partial f_{g}}{\partial x}\right)}^{2}{u^{2}}_{x}+\ldots +{\left(\frac{\partial f_{g}}{\partial x_{s1}}\right)}^{2}{u^{2}}_{x_{s1}}+\ldots +{\left(\frac{\partial f_{g}}{\partial T}\right)}^{2}{u^{2}}_{T}+\ldots +{\left(\frac{\partial f_{g}}{\partial \tau_{14}}\right)}^{2}{u^{2}}_{\tau_{14}}.$$

In Equation (10), the contribution of oil temperature to the combined measurement uncertainty of the algorithm for detecting the location of PD using the non-iterative all-acoustic method is given by (∂*f_g_*/∂*T*)^2^*u*^2^_T_.

According to Equation (10) it is necessary to calculate the sensitivity of the non-iterative algorithm to the transformer oil-temperature change (∂*f_g_*/∂*T*) and standard uncertainty of oil temperature (*u*_T_). The standard uncertainty of oil temperature is calculated by using the statistical method. The sensitivity of the non-iterative algorithm to the transformer oil-temperature change is numerically estimated by using the algorithm for simultaneous variations in input variables that is described in the proceeding chapter.

In the numerical estimation, the maximum value of the change in the PD location result of all three axes is used for the value of the sensitivity of the non-iterative algorithm (Equation (11)).

(11) $${\left(\frac{\partial f_{g}}{\partial T}\right)}_{\max}=\max(\frac{\partial f_{x}}{\partial T},\frac{\partial f_{y}}{\partial T},\frac{\partial f_{z}}{\partial T})\approx \max(\frac{\Delta x}{\Delta T},\frac{\Delta y}{\Delta T},\frac{\Delta z}{\Delta T})=\;\frac{\max(\Delta x,\Delta y,\Delta z)}{\Delta T}=\;\frac{\Delta g_{\max}}{\Delta T}.$$

### 2.4. Measurement Uncertainty Assistant

The MUA software version 1.0 is used to calculate the contribution of oil temperature to the standard combined measurement uncertainty of the non-iterative algorithm. The software implements the algorithm for simultaneous variations in input variables. This algorithm uses the Monte Carlo (MC) method and the one-at-a-time oil temperature algorithm described in [21]. 

**Figure 2:**
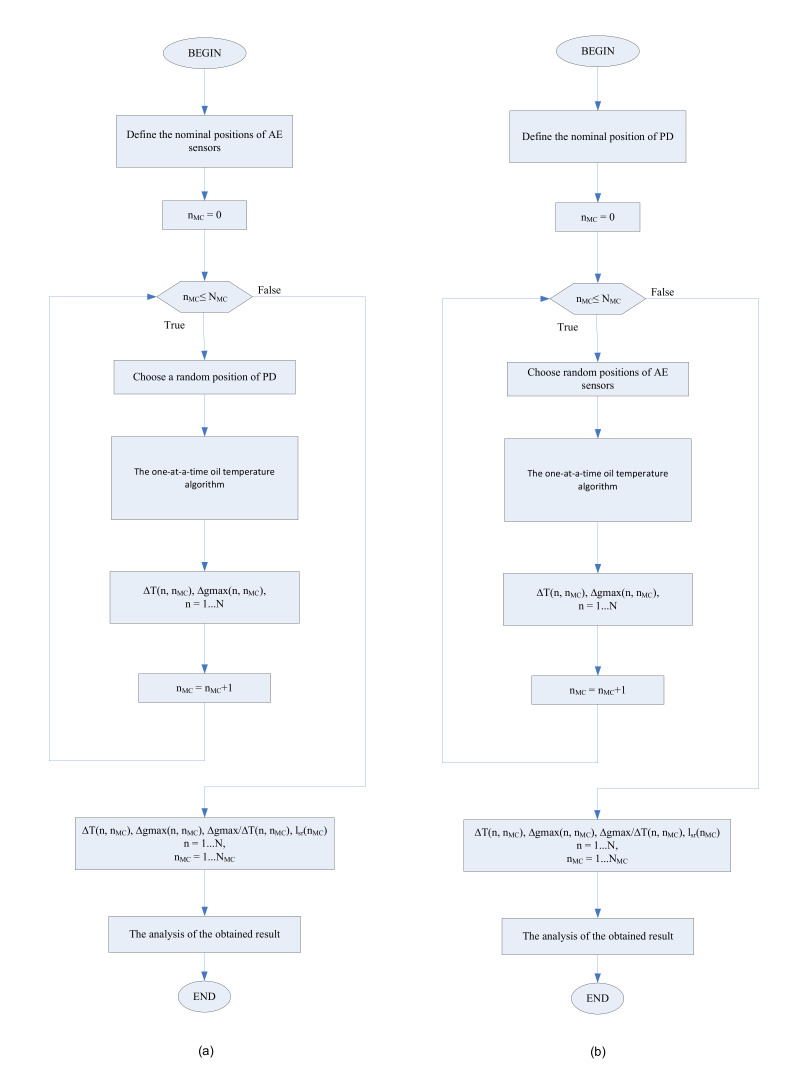
(**a**) First variant of the algorithm for simultaneous variations in input variables, where the positions of the acoustic emission (AE) sensors remain unchanged; (**b**) second variant of the algorithm, where the PD position remains unchanged. The current iteration number is marked with *n*_MC_.

In Figure 2a, the first variant of the algorithm for simultaneous variations in input variables is shown. This variant varies simultaneously with four input parameters: T, x, y, and z. The number of different values used for PD location is marked with N_MC_ (the number of MC simulations).

In Figure 2b, the second variant of the algorithm is shown. This variant varies simultaneously with 13 input parameters: *T*, *x*_Si_, *y*_Si_, and *z*_Si_ (*i* = 1,..., 4). The number of different values used for the AE sensors’ locations is marked with *N*_MC_.

In both variants of the algorithm, the number of different values for the oil temperature used to probe the PD location is marked with *N*. The maximum change in the result to detect the PD location is marked with ∆*g*_max_ = max(∆*x*, ∆*y*, ∆*z*). The position of the PD is described by the mean distance from the AE sensors *l*_sr_ = (*l*_1_ + *l*_2_ + *l*_3_ + *l*_4_)/4. The result is a set of paired values: ∆*T*(*n*, *n*_MC_), ∆*g*max(*n*, *n*_MC_), ∆*g*max/∆*T*(*n*, *n*_MC_), and *l*_sr_(*n*_MC_), where *n* = 1,..., *N* and *n*_MC_ = 1,..., *N*_MC_.

**Figure 3:**
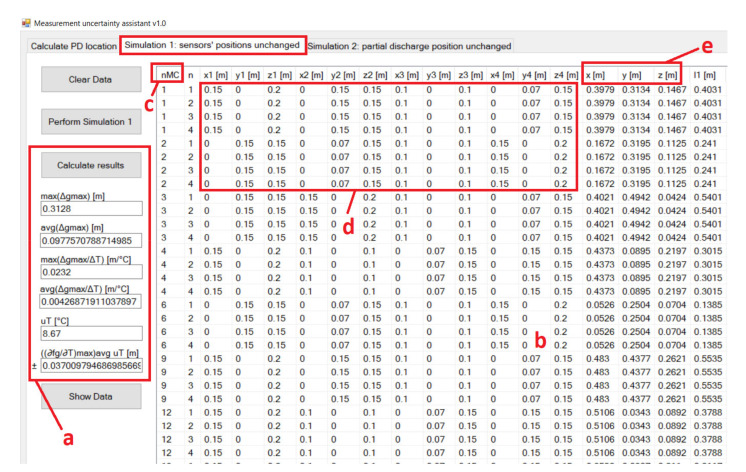
Presentation of the results of the first variant of the algorithm for simultaneous variations in input variables in the MUA software. (**a**) Calculation and display of significant simulation parameters; (**b**) display of valid simulation results and selected columns from the database; (**c**) MC simulation number; (**d**) the sensors’ arrangement is different depending on the PD position; (**e**) random positions of PD.

**Figure 4:**
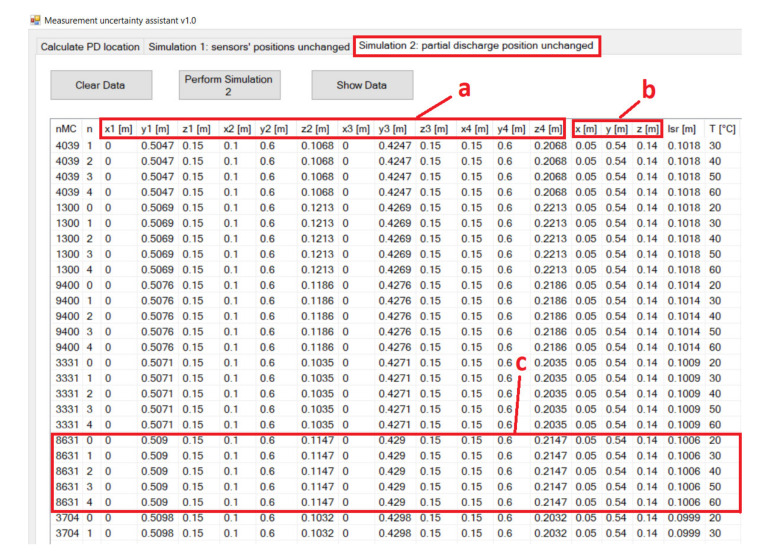
Presentation of the results of the second variant of the algorithm for simultaneous variations in input variables in the MUA software. (**a**) Sensors’ positions are randomized; (**b**) PD location remains unchanged; (**c**) the results of one-at-a-time oil temperature algorithm within the single MC simulation.

The MUA software was written in Visual C# programming language and stored the simulation results in a database. The development tool was Microsoft Visual Studio Community 2019. The user interface of the MUA software displays three tabs. The first tab from left to right is for PD location calculation based on manually inserted input parameters of the non-iterative algorithm. The second and third tab display the significant simulation parameters and valid simulation results of the first and the second simulation, respectively (Figure 3 and Figure 4).

## 3. Results

### 3.1. Simulation Results

In the simulations, a cube-shaped transformer with a side length of 0.60 m was considered. The nominal value for the oil temperature was 20 °C (which was considered the real oil temperature value). Oil temperatures (sound velocities) that were intentionally used instead of the nominal value, while the other nominal input parameters remained unchanged, were: 30 °C (1374 m/s), 40 °C (1337 m/s), 50 °C (1301 m/s), and 60 °C (1266 m/s). The temperature change was marked as *∆T* and took the values of 10 °C, 20 °C, 30 °C, and 40 °C.

**Figure 5:**
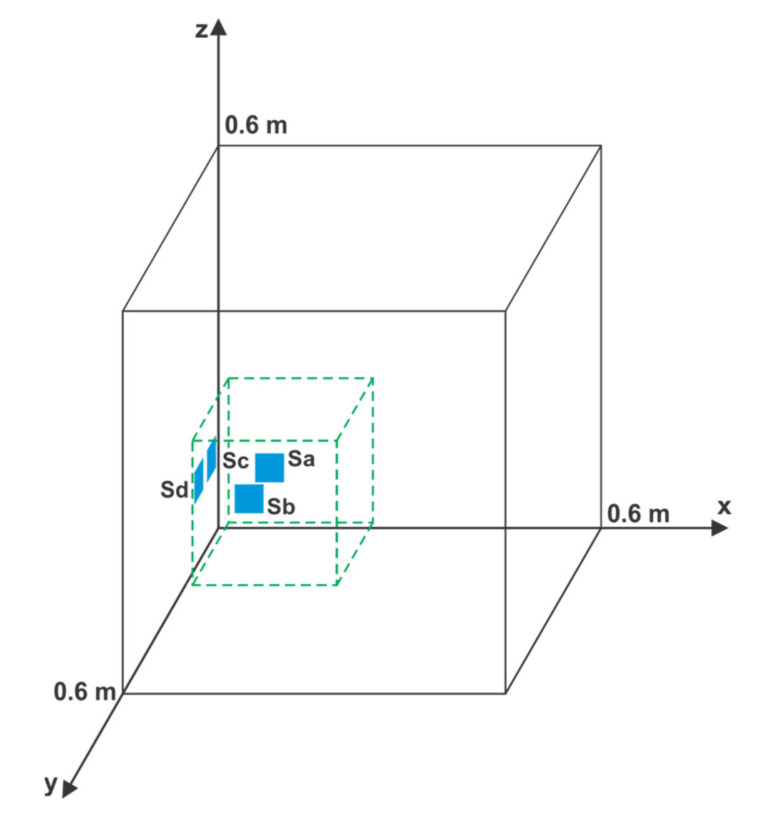
Nominal position of sensors S_a_, S_b_, S_c_, and S_d_; area marked with a dashed-line parallelepiped, which includes PDs with ∆*g*max lower than 0.05 m for a temperature change of 40 °C, in the first simulation.

In the first simulation, the sensor positions were not changed while the PD location was randomized. To illustrate the correlation of the maximum change in the result of detecting the location of PD and mean distance of PD from the AE sensors (*l_sr_*), an example in which the sensors are placed near one edge of the cube-shaped transformer was chosen (Figure 5). Sensor nominal positions are S_a_ (0.15 m, 0.00 m, 0.20 m), S_b_ (0.10 m, 0.00 m, 0.10 m), S_c_ (0.00 m, 0.07 m, 0.15 m), and S_d_ (0.00 m, 0.15 m, 0.15 m). The number of Monte Carlo simulations is *N_MC_* = 10,000.

**Figure 6:**
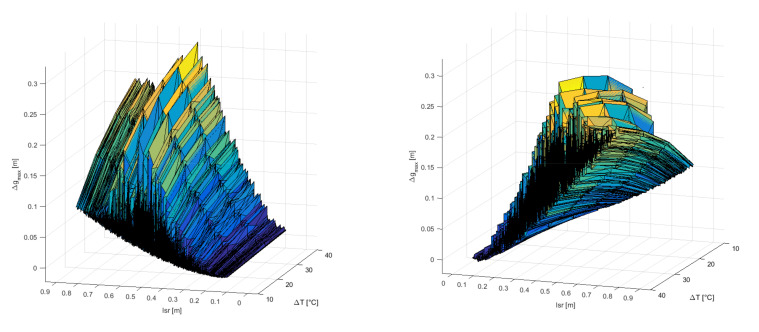
Maximum change in the result for detecting the location of PD ∆*g*max over the mean distance between PD and AE sensor *l*_sr_ and temperature change ∆*T*, in the first simulation.

**Figure 7:**
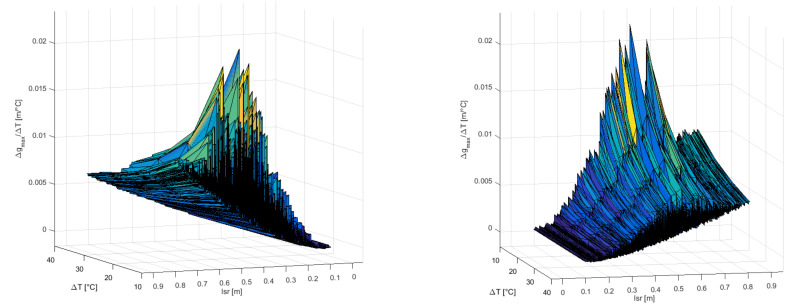
Maximum sensitivity of the algorithm concerning the transformer oil-temperature change ∆*g*max/∆*T* over the mean distance between the PD and AE sensor *l*_sr_ and temperature change ∆*T*, in the first simulation.

For every Monte Carlo simulation, five different temperatures were considered in the algorithm to calculate the effect of the oil-temperature change on the accuracy of PD localization, and the total number of iterations of the algorithm was 50,000. Approximately half of the simulations produced valid results. The results of the simulation are shown in Figure 6 and Figure 7 and include only the valid results and PDs that are distanced from the edges of the cube-shaped transformer by more than 0.03 m.

The maximum change in the results has a maximum value of 0.30 m for *l*_sr_ = 0.51 m and ∆*T* = 40 °C and a minimum value of 0.00 m for *l*_sr_ = 0.12 m and ∆*T* = 10 °C. The average value of ∆*g*max is 0.09 m.

In Figure 6, the maximum change in the result for detecting the location of PD is lower for PD positions with a lower mean distance between PD and AE sensors, even for high-temperature changes. For example, at the PD positions with a mean distance between sensors below 0.20 m, the maximum change in the result is below 0.05 m, even for a temperature change of 40 °C. There are approximately 600 PD positions that meet this criterion, and the area they occupy is marked with a dashed line parallelepiped with side lengths of 0.23 m, 0.22 m, and 0.26 m in Figure 5.

For a temperature change of 10 °C, there are PD positions with a high mean distance from the AE sensors with the maximum change in the result below 0.10 m. That is not the case for a temperature change of 40 °C, where the maximum change in the result increases much faster with an increasing mean distance between the PD and AE sensors.

In Figure 7, the maximum sensitivity of the algorithm with respect to the transformer oil-temperature change, ∆*g*max/∆*T*, over the mean distance between the PD and AE sensors and the temperature change are presented. The maximum sensitivity of the algorithm has a maximum value of 0.0228 m/°C for *l*_sr_ = 0.49 m and ∆*T* = 10 °C. The average value for ∆*g*_max_/∆*T* is 0.0043 m/°C (Table 1).

The contribution of the oil temperature to the combined measurement uncertainty can now be estimated in the first simulation. If it is assumed that the temperature has a discrete uniform distribution, then the standard uncertainty of *T* is *u*_T_ = 15 °C/√3 = 8.67 °C. For the value of ((∂*f_g_*/∂*T*)_max_)_avg_, the estimated average value for ∆*g*max/∆*T* can be used, which is 0.0043 m/°C. Then, the estimation for ((∂*f_g_*/∂*T*)_max_)^2^_avg_ *u*^2^_T_ can be calculated, which is 0.0013 m^2^.

**Figure 8:**
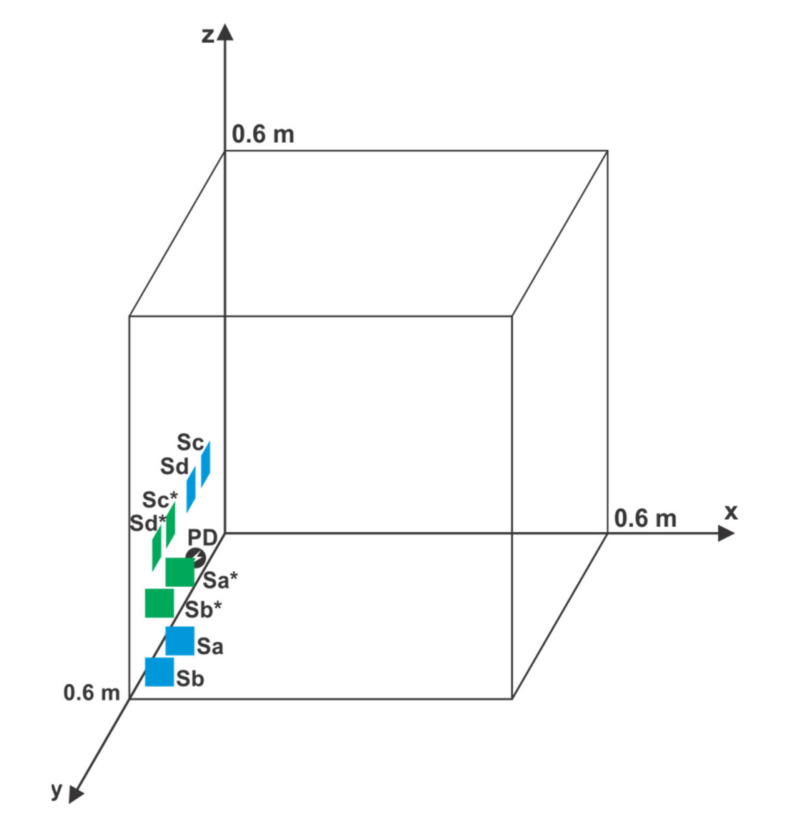
Nominal position of PD, starting position of sensors (S_a_, S_b_, S_c_, and S_d_), and optimal position of sensors (S_a_*, S_b_*, S_c_*, and S_d_*), in the second simulation.

For the second simulation, one PD position from the first simulation, which has a high maximum change in the result for detecting the location of PD, was chosen. For example, the PD position (0.05 m, 0.54 m, 0.14 m), which has a mean distance from the AE sensors of 0.49 m was chosen (Figure 8). For this PD position, the maximum change in the result ranged from 0.19 m for a temperature change of 10 °C to 0.28 m for a temperature change of 40 °C.

**Figure 9:**
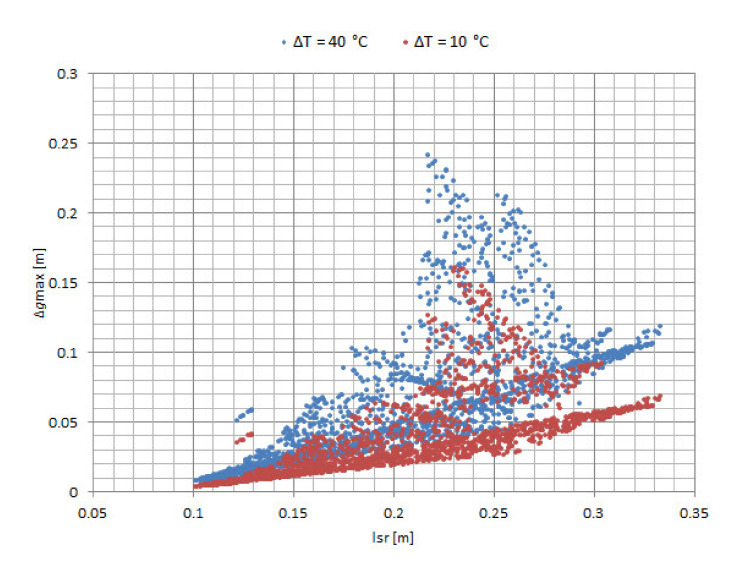
Maximum change in the result for detecting the location of PD ∆*g*max in the second simulation depending on the mean distance between the PD and AE sensor *l*_sr_ for temperature changes (∆*T*) of 10 °C and 40 °C.

Then, the sensor positions were randomized while this PD position remained unchanged. The problem with similarly randomizing sensor positions as in the first simulation is that two prerequisites need to be met while placing the AE sensors. First, sensors should not be close to each other. Second, any symmetry in the positioning of PD and AE sensors should be avoided [15]. To comply with these two prerequisites and avoid the rejection of a large number of individual simulations, similar sensor positions to those of the first simulation as the starting positions of randomization are used; these sensor positions are S_a_ (0.15 m, 0.60 m, 0.20 m), S_b_ (0.10 m, 0.60 m, 0.10 m), S_c_ (0.00 m, 0.07 m, 0.15 m), and S_d_ (0.00 m, 0.15 m, 0.15 m). The difference from the first simulation is that sensors S_a_ and S_b_ are now placed on the opposite wall of the cube-shaped transformer (Figure 8). Then, the positions of S_a_ and S_b_ were randomized along the *z* axis by keeping the relative position of the sensors unchanged and independently for S_c_ and S_d_ along the *y* axis. The number of performed Monte Carlo simulations was *N*_MC_ = 10,000. The results of the simulations are presented in Figure 9.

For example, in Figure 9, for sensor positions with *l*_sr_ of 0.15 m, the maximum change in the result for detecting the PD location ranges from 0.01 m for a temperature change of 10 °C to 0.04 m for a temperature change of 40 °C. This outcome is a significant improvement over the first simulation, accomplished by bringing the AE sensors closer to the PD location. In Figure 8, this action is illustrated with the depiction of the starting positions of the sensors and the new (optimal) sensor positions: S_a_*(0.15 m, 0.60 m, 0.24 m), S_b_*(0.10 m, 0.60 m, 0.14 m), S_c_*(0.00 m, 0.32 m, 0.15 m), and S_d_*(0.00 m, 0.40 m, 0.15 m). For an optimal position, the maximum change in the result for detecting the location of PD ranges from 0.01 m for a temperature change of 10 °C to 0.03 m for a temperature change of 40 °C.

Then, the contributions of the oil temperature to the combined measurement uncertainty in the second simulation were compared. For the same sensor positions as those in the first simulation, the average value of ∆*g*max/∆*T* was 0.0115 m/°C, leading to an estimation for *(∂f_g_/∂T*)^2^_max_ *u*^2^_T_ of 0.0099 m^2^, which was approximately ±0.10 m for the value of *(∂f_g_/∂T*)_max_ *u*_T_. For the optimal placement of the sensors, the average value for ∆*g*max/∆*T* was 0.0011 m/°C with the estimation for *(∂f_g_/∂T*)^2^_max_ *u*^2^_T_ of 0.0001 m^2^, which was ±0.01 m for the value of *(∂f_g_/∂T*)_max_ *u*_T_. The estimated value for *(∂f_g_/∂T*)^2^_max_ *u*^2^_T_ in the first case was approximately 100 times greater than that in the second case (Table 2).

### 3.2. Experimental Results

**Figure 10:**
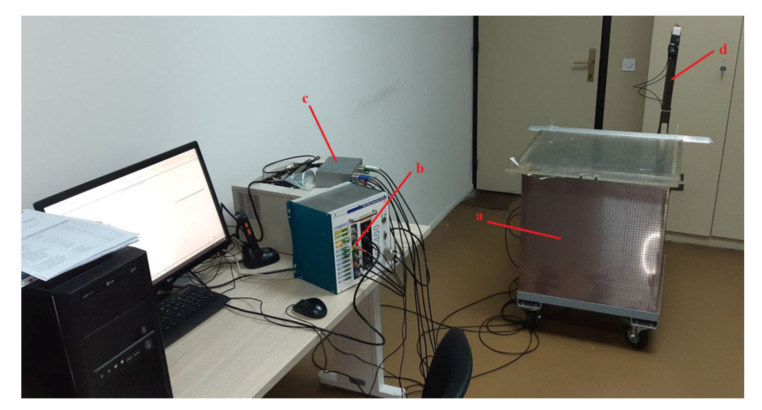
Photograph of the experimental setup: (**a**) stainless steel tank filled with transformer oil; (**b**) Picoscope 5444D oscilloscope card; (**c**) conditioning circuitry; and (**d**) PD spark source attached to the end of the metal meter immersed in the transformer oil.

**Figure 11:**
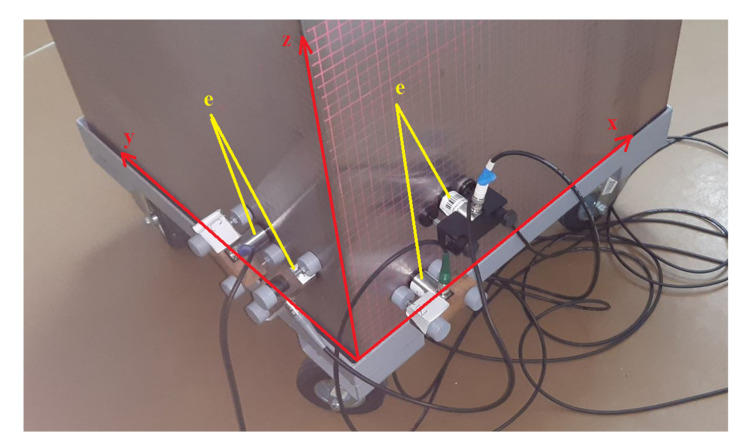
Photograph of the four Power Diagnostix AS 75l sensors mounted on the tank wall; the sensors were coupled to the tank’s wall, applying oil (**e**); the assumed directions of axes *x*, *y*, and *z* are also indicated.

**Figure 12:**
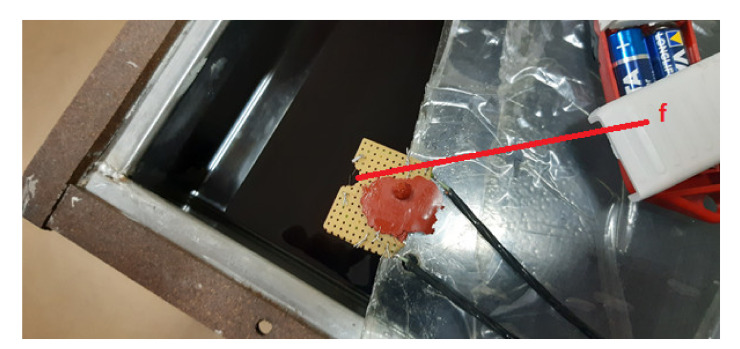
Photograph of the PD corona spark source (**f**).

The experiments were performed at the Institute of Nikola Tesla, Belgrade, Serbia. The experimental setup consisted of a stainless steel tank (0.60 m, 0.60 m, 0.65 m) filled with mineral transformer oil, a PC equipped with a Picoscope 5444D Pico Technology, St Neots, United Kingdom, quad-channel oscilloscope card, a conditioning circuitry, four Power Diagnostix AS 75l Power Diagnostix Systems GmbH, Aachen, Germany, piezo-electric ultrasound sensors, and a PD corona spark source that was attached to the end of a metal meter for convenience (Figure 10, Figure 11 and Figure 12).

Arrival time (AT) is the time at which the AE signal arrives at a sensor. The AE PD signal is complex because of various propagation routes, refraction, and reflection. The signal travels to the sensor through different propagation mediums like transformer oil, tank walls, cores, windings, etc. There are three typical AE signal travel paths. A straight-line path of a direct signal through the shortest route from PD to the sensor. An indirect signal path along the tank wall through which AE signal arrives at the sensor earlier than the direct signal because the AE signal’s velocity in metal is significantly higher than in the oil. A reflected signal path from signals reflected from structures such as cores, windings, and tank walls that propagate longer and slower. The direct signal has greater energy and higher amplitude, and it is used by most of the researchers for AT. Because of possibility of total reflection of the direct signal, some researchers recommend using the indirect signal instead [32]. In noisy environments with weak PD signals, the appropriate denoising techniques are employed before AT determination [33].

In the experiments, the AT was hand-picked. The hand-picking AT method may be used in experiments but is impractical for applications in the field [32]. The amplitude of the noise was relatively small. The indirect signal amplitude was relatively small compared to the amplitude of the direct signal. The arrival of a stronger direct PD signal was noticeable. The direct signal consisted of major pulses from which the first significant negative pulse was chosen as the referent pulse.

The experiment began by placing the four sensors in their nominal positions, which were the same as those in the first simulation (Figure 5). The temperature of the transformer oil was approximately 20 °C. The PD source was placed in ten different positions, which were chosen within the area marked with the dashed-line parallelepiped in Figure 5 (∆*g*max < 0.05 m). For every PD position, ten measurements of TDOAs were performed, and their average values and standard deviations were calculated. Subsequently, the values of ∆*g*max in the appropriate temperature change function were calculated (Table 3).

**Figure 13:**
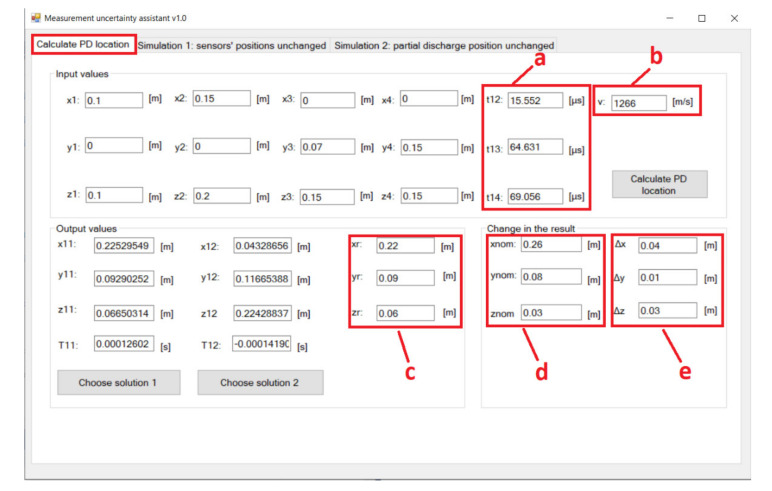
Using MUA software to determine ∆*g*max for PD position 8 in Table 3. (**a**) Measured average values of TDOAs while temperature of the transformer oil was 20 °C; (**b**) the incorrect sound velocity value that corresponds to transformer oil temperature of 60 °C; (**c**) calculated PD location using the incorrect value for sound velocity; (**d**) calculated PD location using the correct value for sound velocity (the nominal value of 20 °C); (**e**) change in the result over the axes (∆*g*max = ∆x = 0.04 m).

The results in Table 3 show that the values for ∆*g*max varied from 0.00 m to 0.04 m for the PD positions and oil-temperature changes under study. This indicates that when the real transformer oil temperature was approximately 20 °C, if it is (incorrectly) assumed that the temperature was, for example, 60 °C (∆*T* = 40 °C), the maximum ∆*g*max during PD position calculation was 0.04 m (Figure 13).

These results are in agreement with those of the first simulation and demonstrate that the fluctuation of the results in the PD location decreases when the PD is placed close to the sensors (lower values of *lsr*).

Afterward, the results of Simulation 2 were tested. The PD was placed in the position depicted in Figure 8, whereas the sensors’ position remained unchanged. Then, after the measurements, the sensors were moved to the optimal position. For both sensor locations, ten measurements of TDOAs were performed, and their average values and standard deviations were calculated; the results are shown in Table 4.

The results in Table 4 reveal that the values of ∆gmax/∆T changed from 0.0083 m/°C for the sensors placed as in the first simulation to 0.0006 m/°C for the sensors in the optimal position. These results are in agreement with those of the second simulation and demonstrate that a significant improvement in the contribution of oil temperature to the combined measurement uncertainty can be achieved by placing the sensors in an optimal position (lower values of *lsr*).

## 4. Discussion

The contribution of oil temperature to the combined measurement uncertainty can be estimated quantitatively using the proposed procedure in uncertainty calculations and the appropriate software tool. The contribution can be used, for example, to complement the recommendation from IEEE C.57.127-2007.

Regarding the contribution, for the placement of specific sensors, there are more and less optimal PD positions (and vice versa) in the cube-shaped transformer considered. The second simulation results demonstrated the significant improvement by one order of magnitude in the contribution of oil temperature to the combined measurement uncertainty from the starting position of the AE sensors to the optimal position of the AE sensors.

The contribution is lower when AE sensors are placed close to the PD (lower values of the mean distance between PD and AE sensors). This placement method for AE sensors is also favorable for AE signal detection (attenuation).

**Figure 14:**
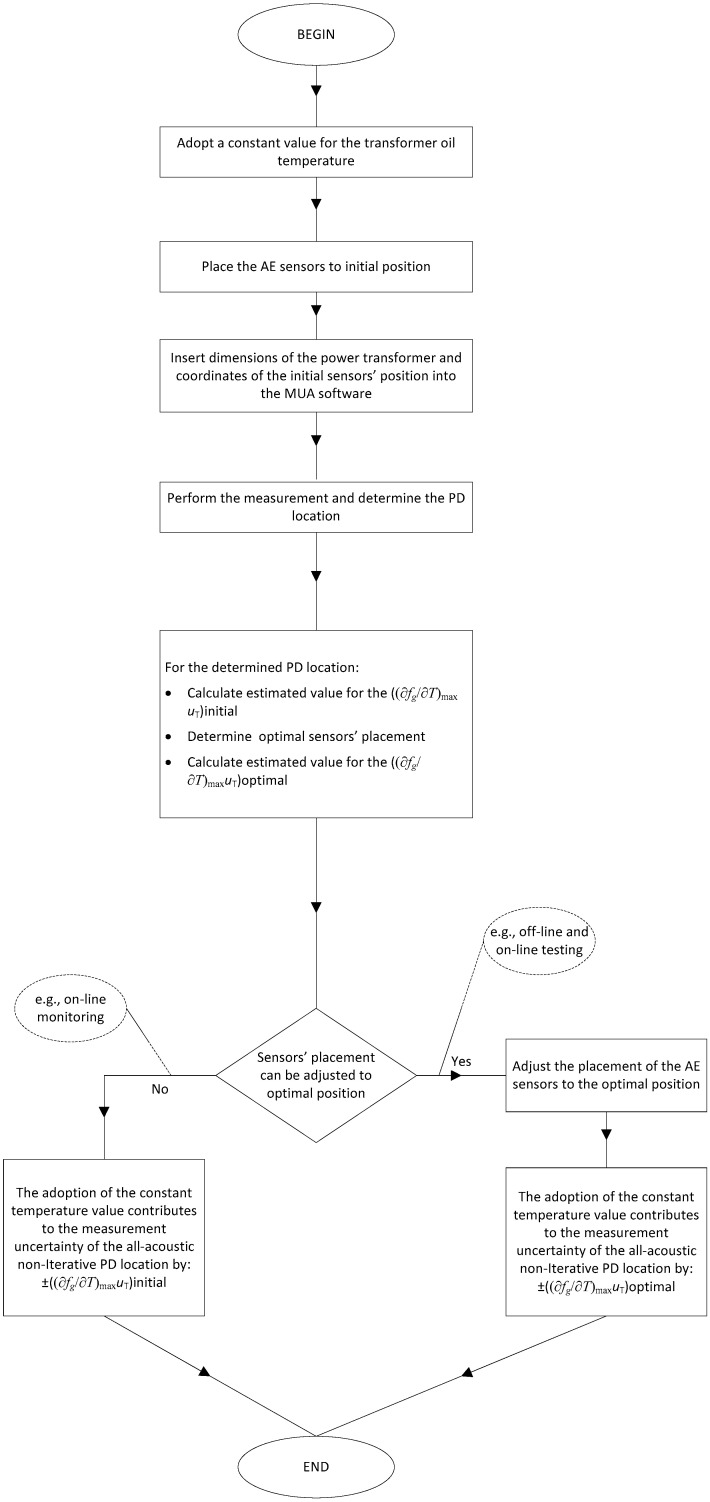
The proposed procedure for quantifying and controlling the contribution of oil temperature to the combined measurement uncertainty of the non-iterative algorithm using the MUA software. The result of the procedure is the quantified contribution of oil temperature to the measurement uncertainty of the method due to the assumption of the constant temperature value. It is assumed that PD is located in the transformer oil.

Based on these findings, Figure 14 shows the proposed procedure for using the MUA software in the practice.

The first step of the proposed procedure is to adopt a constant value for the transformer oil temperature (e.g., based on the literature recommendations). Next, it is necessary to insert the adopted value and the values of the power transformer’s dimensions and the coordinates of the initial sensors’ position into the MUA software and calculate PD location. For the PD position, the estimated initial and optimal values for the contribution of oil temperature to the method’s measurement uncertainty are determined. If the sensors’ placement is adjusted to the optimal position, the appropriate optimal contribution is used. Otherwise, the initial contribution is used. In both cases, the adopted constant value proposed at the beginning of the procedure is accompanied by the associated amount of contribution to the method’s measurement uncertainty. The procedure can be used by the experts performing on-line and off-line power transformer testing. It can also be automated and used by the appropriate monitoring systems.

Controlling and quantifying the contribution of oil temperature to the combined measurement uncertainty of the non-iterative algorithm improves the reliability of the result of PD location. It also enhances the confidence that can be placed in power transformer maintenance decisions based on its use.

The future work will focus on validating the presented findings under field conditions and comparing the obtained results with other PD localization methods. The influence of the variations in the AE signal velocity induced by direct AE signal propagation through materials other than transformer oil on the all-acoustic non-iterative PD localization accuracy will be researched. The effect of variations in the oil temperature on the accuracy of the non-iterative combined acoustic-electrical PD localization will also be researched.

## Figures and Tables

**Table 1:** Estimated contribution of the oil temperature to the combined measurement uncertainty of the non-iterative algorithm, in the first simulation.

Variable	Value	lsr (m)	∆T (°C)
**max(∆gmax) (m)**	0.30	0.51	40
**min(∆gmax) (m)**	0.00	0.12	10
**avg(∆gmax) (m)**	0.09	–	–
**max(∆gmax/∆T) (m/°C)**	0.0228	0.49	10
**avg(∆gmax/∆T) (m/°C)**	0.0043	–	–
***u*** **_T_** **(** **°C** **)**	8.67	–	–
**((∂*f_g_*/∂*T*)_max_) ^2^_avg_*u*^2^_T_ (m^2^)**	0.0013	–	–
**((∂*f_g_*/∂*T*)_max_)_avg_*u*_T_ (m)**	± 0.03	–	–

**Table 2:** The comparison of the contributions of the oil temperature to the combined measurement uncertainty in the second simulation. The improvement by one order of magnitude in the contribution from the starting position of the AE sensors to the optimal position.

Sensors’ Position	lsr (m)	∆gmax(∆T) (m)		Avg(∆gmax/∆T) (m/°C)	(∂f_g_/∂T) ^2^_max_ u^2^_T_ (m^2^)	(∂f_g_/∂T)_max_ u_T_ (m)
–	–	10 °C	40 °C	–	–	–
**As in Simulation 1**	0.49	0.19	0.28	0.0115	0.0099	±0.10
**Optimal**	0.15	0.01	0.03	0.0011	0.0001	±0.01

**Table 3:** Experimental verification of the results of Simulation 1. The results show that, for the PDs placed close to the sensors, ∆gmax remains below 0.05m even for oil-temperature change of 40 °C.

PD Position	Nominal PD Coordinates (m)			Average Values of TDOAs (µs)			Standard Deviations of TDOAs (µs)			∆gmax(∆T) (m)			
	x	y	z	τ12	τ13	τ14	σ12	σ13	σ14	10 °C	20 °C	30 °C	40 °C
**1**	0.06	0.08	0.15	15.30	33.76	47.90	0.30	1.66	1.97	0.00	0.01	0.01	0.01
**2**	0.06	0.18	0.15	39.44	83.66	92.05	1.18	2.99	3.74	0.01	0.01	0.01	0.01
**3**	0.18	0.19	0.14	11.28	20.43	23.25	0.08	0.14	0.42	0.01	0.01	0.01	0.02
**4**	0.17	0.09	0.14	11.61	46.10	52.16	0.10	0.13	0.07	0.00	0.01	0.01	0.01
**5**	0.05	0.18	0.08	35.89	69.37	103.75	0.18	0.24	0.22	0.00	0.01	0.01	0.01
**6**	0.22	0.19	0.07	6.58	9.04	25.28	0.28	0.18	0.10	0.01	0.01	0.02	0.02
**7**	0.10	0.13	0.07	6.62	9.66	44.27	0.17	0.09	0.18	0.00	0.01	0.01	0.01
**8**	0.26	0.08	0.03	15.55	64.63	69.05	0.82	3.13	4.84	0.01	0.02	0.03	0.04
**9**	0.14	0.08	0.07	38.57	43.84	52.20	2.33	2.81	3.81	0.01	0.01	0.01	0.01
**10**	0.07	0.07	0.06	21.80	36.03	60.20	0.89	0.88	0.73	0.01	0.01	0.01	0.01

**Table 4:** Experimental verification of the results of Simulation 2. Significant improvement in the non-iterative algorithm’s sensitivity to the transformer oil temperature achieved by placing the sensors in the optimal position.

Sensors’ Position	Average Values of TDOAs (µs)			Standard Deviations of TDOAs (µs)			∆gmax(∆T) (m)				Average ∆gmax/∆T (m/°C)
–	τ12	τ13	τ14	σ12	σ13	σ14	10 °C	20 °C	30 °C	40 °C	–
**As in Simulation 1**	55.70	104.56	108.34	0.30	0.36	0.42	0.13	0.17	0.20	0.21	0.0083
**Optimal**	18.10	32.99	81.88	0.31	1.47	1.27	0.01	0.01	0.02	0.02	0.0006

## Data Availability

The simulation and experimental data presented in this study are available in FigShare at https://doi.org/10.6084/m9.figshare.14199278 accessed on 11 March 2021. All other data presented in the study are available on reasonable request from the corresponding author.
